# “A cure might help, but it won't erase it all”: a qualitative study of policy challenges and priorities for long‐term survivors of HIV in the United States

**DOI:** 10.1002/jia2.70006

**Published:** 2025-07-03

**Authors:** Ali Ahmed, Jeff Taylor, Rachel Lau, Joyce Ching‐Jung Lai, Sithara Deshan Diunugala, Michael Louella, Thomas J. Villa, William Freshwater, Dawn Averitt, Maile Karris, Jeff Berry, Lynda Dee, Karine Dubé

**Affiliations:** ^1^ Division of Infectious Diseases and Global Public Health School of Medicine University of California San Diego (UCSD) La Jolla California USA; ^2^ HIV + Aging Research Project – Palm Springs (HARP‐PS) Palm Springs California USA; ^3^ Reversing Immune Dysfunction for HIV‐1 (RID‐HIV) Eradication Martin Delaney Collaboratory Community Engagement Coordination and Community Advisory Board San Diego California USA; ^4^ Long‐Term Survivor of HIV, Nationawide USA; ^5^ University of Washington Center for AIDS Research (CFAR) Seattle Washington USA; ^6^ Delaney AIDS Research Enterprise (DARE) Martin Delaney Collaboratory Community Engagement Coordination and Community Advisory Board San Francisco California USA; ^7^ HIV Obstruction by Programmed Epigenetics (HOPE) Delaney Collaboratory Community Advisory Board Gladstone Institutes San Francisco California USA; ^8^ AIDS Law Project of Pennsylvania Philadelphia Pennsylvania USA; ^9^ The Well Project and Women's Research Initiative on HIV/AIDS (WRI) Brooklyn New York USA; ^10^ CRISPR for Cure Martin Delaney Collaboratory Community Advisory Board Philadelphia Pennsylvania USA; ^11^ The Reunion Project Calabasas California USA; ^12^ AIDS Action Baltimore Baltimore Maryland USA

**Keywords:** ageing, HIV cure research, long‐term survivors, people with HIV, policy priorities, socio‐behavioural research, United States

## Abstract

**Introduction:**

Long‐term survivors (LTS) of HIV, including individuals diagnosed before the availability of effective antiretroviral therapy (ART), have played a pivotal role in shaping the HIV response. Despite an increase in their number in the United States, their unique medical, social and economic challenges remain underrepresented in HIV policy and research, particularly in the context of HIV cure advancements. While an HIV cure may alleviate ART‐related burdens, LTS fear unintended consequences, including the potential loss of critical social benefits, economic support and healthcare access. This study explores the policy priorities of LTS, addressing their current unmet needs and the broader implications of an HIV cure.

**Methods:**

We conducted qualitative interviews with 32 LTS across diverse racial, gender and geographic backgrounds, recruited through community‐based organizations and research networks from 2023 to 2024. Using inductive thematic analysis, we identified key policy concerns and recommendations based on participants’ lived experiences. Data collection continued until thematic saturation was reached.

**Results:**

LTS emphasized four pressing policy domains: (1) Persistent Healthcare Disparities: Participants reported fragmented Medicare and Medicaid coverage, limited access to essential services (e.g. dental, vision and mental healthcare), and ongoing stigma and discrimination in healthcare settings. (2) Social and Economic Precarity: Housing instability, financial insecurity and employment barriers disproportionately affect LTS, many of whom face systemic barriers to re‐entering the workforce. (3) Policy Implications of an HIV Cure: Participants voiced concerns that an HIV cure, while promising, could result in disqualification from disability and social assistance programmes, exacerbating socio‐economic vulnerabilities. (4) Structural Reforms for LTS Inclusion: LTS underscored the urgent need for their direct involvement in HIV research, policy development and decision‐making to ensure equitable, community‐driven solutions.

**Conclusions:**

Policymakers must address comprehensive healthcare access, economic stability and social protections for LTS of HIV. HIV cure research must not undermine existing benefits or widen disparities. Ensuring LTS representation in decision‐making is critical to developing equitable policies that safeguard their wellbeing before and after a cure.

## 1 INTRODUCTION

In the United States, nearly half of the people living with HIV (PLWH) are aged 50 years or older, reflecting a growing population of individuals who have lived with HIV for a decade or more, commonly referred to as long‐term survivors (LTS) [1, 2]. LTS are often defined as individuals diagnosed before widespread access to effective antiretroviral therapy (ART), or those living with HIV for more than 10 years [1]. They have experienced the profound shifts in HIV care, stigma and survival that have marked the epidemic over time [1]. LTS can provide critical insights into the evolving landscape of HIV treatment, care, research and policy. Though ART transformed HIV into a manageable chronic condition, LTS now face complex ageing‐related challenges [3]. Accelerated biological ageing, cumulative comorbidities and the lingering effects of early, often toxic, treatment regimens disproportionately affect this group [4, 5]. Despite their vital role in shaping the HIV response, the perspectives and priorities of LTS remain underrepresented in research and policy frameworks, particularly in the context of HIV cure research efforts, which encompass scientific studies and clinical trials aimed at achieving durable ART‐free control or complete elimination of HIV from the body. This exclusion represents a critical gap that this study seeks to illuminate.

Existing literature has extensively documented the medical and psychosocial burdens of ageing with HIV, including higher risks of cardiovascular disease, diabetes, osteoporosis, neurological complications and mental health challenges [6, 7]. However, these studies often fail to situate these challenges within the broader socio‐economic and political realities of being an LTS in the United States [8]. Many LTS contend with housing insecurity, inadequate healthcare access and social isolation issues exacerbated by socio‐economic inequities and enduring HIV stigma combined with ageism [9, 10]. Moreover, policy implications remain underexplored, with little attention to how support structures must evolve as HIV cure research advances.

Accelerating momentum in HIV cure research [11] introduces both opportunities and uncertainties for LTS. A cure may ease ART burdens and reduce stigma [12, 13], but could bring significant personal and systemic costs. Many LTS rely on public programmes such as *Medicare*, a U.S. federally funded health insurance programme for people aged 65 and older and certain younger individuals with disabilities; *Medicaid*, a state‐administered programme that provides health coverage to low‐income individuals and families, jointly funded by state and federal governments; and the *Ryan White HIV/AIDS Program*, a federally funded initiative that provides medical care and essential support services to uninsured or underinsured PLWH. Additional supports include the *AIDS Drug Assistance Program* (ADAP), which helps cover the cost of HIV medications, and housing support through the *Housing Opportunities for Persons With AIDS* (HOPWA), which offers partially or fully subsidized housing based on income eligibility and programme availability [14, 15]. An HIV cure could potentially reclassify individuals as no longer living with HIV, jeopardizing their access to essential services. Further, the prospect of losing an HIV identity central to many LTS communities raises critical psychosocial considerations that current health and social systems are ill‐equipped to address [16, 17]. These complexities highlight the urgent need for proactive strategies to avoid unintended consequences for ageing PLWH.

Equally pressing is the limited inclusion of LTS in HIV cure research and related policy frameworks, despite making up a significant portion of the ageing population [1]. With decades of lived expertise navigating the complexities of HIV treatment, stigma and ageing, LTS are uniquely positioned to identify actionable policy gaps. Yet, many remain excluded from trials and decisions [2, 18], limiting equity and exacerbating disparities among racially minoritized or geographically isolated LTS [19]. This study identifies key policy priorities from the perspectives of LTS, examining both their current needs and the additional risks posed by HIV cure research. Although existing policies address healthcare, housing and social support, cure‐related advances may disrupt these frameworks. For example, Timothy Ray Brown, the first person cured of HIV, lost housing benefits following his cure [14], highlighting the urgent need for protective and transitional policies. By centring the voices of LTS, this study aims to identify policy priorities that ensure continuity of care and support during and after HIV cure research. We focused on LTS due to their long experience and distinct needs compared to more recently diagnosed individuals, particularly regarding long‐term service access, cumulative stigma and systemic structural inequities. A forward‐looking, LTS‐informed policy agenda is essential to safeguard their rights and promote equity in future HIV cure research.

## 2 METHODS

### 2.1 Study setting and participants

For this study, we defined LTS as individuals who had been living with HIV for 10 or more years. We included participants who were aged 60 or older, had lived with HIV for at least 10 years, resided in the United States, spoke English and had experience in HIV advocacy, peer support, or research engagement. We conducted a brief screening process to confirm eligibility prior to enrolment. We enrolled a total of 32 LTS from across the United States using a customized, non‐probabilistic sampling approach designed to capture diversity across sex, gender, race and ethnicity. We recruited participants through outreach efforts with community partners affiliated with three Martin Delaney Collaboratories for HIV Cure Research: the Delaney AIDS Research Enterprise (DARE) [LD, ML, JT], Reversing Immune Dysfunction for HIV‐1 Eradication (RID‐HIV) [JT, ML, TJV] and CRISPR for Cure [JB]. We also collaborated with three community‐based organizations (CBOs): AIDS Action Baltimore [LD], the HIV + Aging Research Project—Palm Springs (HARP‐PS) [JT], the Reunion Project [JB] and additional CBOs focused on ageing and HIV, including NMAC (formerly the National Minority AIDS Council), The Well Project and the San Francisco AIDS Foundation (SFAF). Investigators and clinicians specializing in HIV and ageing also helped with recruitment. We used an institutional review board (IRB)‐approved flyer to increase awareness and encourage participation (available upon request to the corresponding author).

### 2.2 Study design

We chose a qualitative design to explore the policy priorities of LTS of HIV because it enabled the capture of rich, detailed narratives that quantitative methods cannot [20]. This approach allowed us to capture their values, concerns and policy priorities, and to develop a participant‐informed agenda grounded in the realities of ageing with HIV [21].

### 2.3 Interview guide

The research team developed the interview guide in consultation with LTS, including LTS contributors to this manuscript, to ensure it reflected lived experiences, enthusiasm and concerns about HIV cure research and policy priorities. Prompts encouraged participants to discuss issues like the potential loss of HIV identity, changes to medical and support services, and socio‐economic impacts in areas such as Medicaid/Medicare, disability, housing benefits and Ryan White support services (Table S1).

### 2.4 Data collection

The study's principal investigator (KD) scheduled interviews with participants after they expressed interest in participating. KD provided each participant with an IRB‐approved informed consent form, a demographic questionnaire and an interview guide. Two research team members (JT and KD) conducted and recorded all interviews in English using teleconference (Zoom), a virtual conferencing platform compliant with the Health Insurance Portability and Accountability Act (HIPAA) regulations. Upon completing the interview, participants received a $50 Visa gift card as compensation, delivered either by mail or electronically.

### 2.5 Data analysis

Upon interview completion, the research team saved the audio files on a secure drive that only they could access. The team transcribed verbatim each file using an encrypted transcription service. Research assistants (RL, JC‐JL and SDD) reviewed and corrected each transcript against audio recordings and de‐identified transcripts in the process. Due to the exploratory nature of the study, we chose conventional thematic analysis with inductive reasoning to analyse the data [22]. The research team then compiled each participant's response to the associated question in a master transcript document for easier visualization when the data were manually coded.

Two team members (AA and KD) double‐coded the data using Microsoft Word and Excel. We did not use a pre‐existing coding scheme due to the novelty of this research. The primary coder was a post‐doctoral researcher (AA) who created the initial codebook, extracted key quotes and generated tables. KD served as the secondary coder by reviewing and refining the initial coding. We organized the quotes in a table format and resolved discrepancies through discussion and agreement during bi‐weekly virtual meetings. After an extensive review of the transcripts, we reached thematic saturation [23]. The co‐authors validated the emerging themes and approved the final themes during manuscript preparation. Consistent with reflexive and interpretive qualitative methods [24], we did not calculate inter‐coder agreement metrics, as our emphasis was on collaborative interpretation and consensus‐building rather than statistical concordance.

### 2.6 Ethics statement

We conducted this study in line with U.S. Federal Regulations and the Declaration of Helsinki. The University of California San Diego (UCSD) IRB approved the study (IRB #808882). Participants provided their IRB‐approved verbal consent, which was recorded on their audio file. We provided each participant with the informed consent form before their interview. We de‐identified all study‐related documents and transcripts and destroyed all audio files after transcription review to ensure confidentiality.

## 3 RESULTS

### 3.1 Study participants

We recruited 32 LTS, with a mean age of 66.6 years (median 69), and participants had lived with HIV for an average of 33 years. The sample was diverse, with 50% identifying as cisgender men, 46.9% as cisgender women and 3.1% as transgender women. Educational attainment varied, with 43.7% holding a college degree, 37.5% having some college education and 6.3% holding a master's degree. Racially, 53.1% identified as White, 37.5% as Black, and 6.2% each as Asian and mixed race. Geographically, 53.1% resided in the West, 25% in the South, 18.8% in the Northeast and 3.1% in the Midwest. Participants reported an average of 27.4 years of HIV advocacy, with 7.5 years specifically focused on HIV cure research (Table 1).

**Table 1:** Demographic characteristics of long‐term survivor participants (*n* = 32; United States, 2023–2024)

Characteristics	*N* (%)
Age in years, mean (min‐max)	66.6 (60–82)
Sex assigned at birth	
Male	15 (46.9)
Female	17 (53.1)
Gender	
Cisgender man	16 (50.0)
Cisgender woman	15 (46.9)
Transgender woman	1 (3.1)
Education	
Less than high school	1 (3.1)
High school diploma or GED	2 (6.3)
Some college, but no diploma	12 (37.5)
College graduate	14 (43.7)
Master's degree or equivalent	2 (6.3)
Doctorate degree or equivalent	1 (3.1)
Race	
White/Caucasian	17 (53.1)
Black/African American	12 (37.6)
Indian	1 (3.1)
Filipino	1 (3.1)
Mixed	1 (3.1)
U.S. region	
Northeast	6 (18.8)
Midwest	1 (3.1)
South	8 (25.0)
West	17 (53.1)
Years worked in HIV‐related field, mean (std. dev.)	27.4 (9.39)
Years involved in HIV cure research, mean (std. dev.)	7.5 (10.43)
0 to <1	15 (46.9)
1−10	11 (34.3)
11−20	1 (3.1)
21−30	3 (9.4)
31−40	2 (6.3)

Abbreviation: GED, general educational development.

Results are structured around four overarching themes: (1) health policy challenges (pre‐cure); (2) social policies and benefits (pre‐cure); (3) policy implications in the context of HIV cure (post‐cure); and (4) broader structural reforms and future policy directions. Table S2 provides additional quotes that emerged from the below themes.

### 3.2 Health policy challenges (pre‐cure)

#### 3.2.1 Healthcare tailored to LTS

LTS advocated for policies that recognize their unique, complex health challenges after decades of living with HIV. They emphasized the need for a holistic approach to care that considers the intersecting effects of prolonged ART use, ageing and comorbidities.

Healthcare for LTS is not the same as for someone newly diagnosed… We're dealing with decades of medications, aging, and other issues. The system needs to catch up to the fact that our needs are different, and policies should reflect that.—Male, Black

LTS emphasized the importance of policies designed specifically for LTS, acknowledging the impact on overall health and wellbeing, career and education, and ageing trajectory for people who have lived with HIV for many years.

#### 3.2.2 Complex healthcare needs and inadequate coverage

LTS face persistent challenges navigating disjointed Medicare and Medicaid services, particularly in securing access to essential dental and vision care, which remain prohibitively expensive and poorly covered.

Dental care is a nightmare for people like us. Medicaid doesn't cover most of what we need, and it's hard to find dentists who'll take us. I've lost teeth because I couldn't afford the treatments. Vision care is just as bad. How are we supposed to live healthy lives if we can't even get the basics taken care of?—Male, Black

High copays further exacerbate financial burdens, with some participants reporting hundreds of dollars in out‐of‐pocket costs for necessary services each month.

Every time I go to see a doctor; it's a $40 copay… In the month of October… I did $650 worth of copay. Because I had my mental health visits, physical therapy, and various specialists. If I don't have ADAP to at least pay my health insurance, now I'm really screwed.—Male, Black

While programmes like ADAP provide critical support, many LTS struggle to navigate complex healthcare systems or are forced to forgo care due to restrictive coverage policies. Participants emphasized that existing safety nets, designed during a time when long‐term survival was not anticipated, are ill‐equipped and severely underfunded to address the realities of ageing with HIV.

#### 3.2.3 Mental health support

LTS emphasized the critical importance of access to culturally responsive mental health in HIV policies and care frameworks. They highlighted the profound compounding psychological toll of living with HIV, stemming from loss, discrimination and trauma, that has remained inadequately addressed.

We've lost so many friends, faced so much discrimination, and lived with the constant fear of dying. It takes a toll on you. I've had therapy over the years, but it's not enough. Policies need to make mental health a priority, not an afterthought.—Male, White

Participants also stressed the need for every HIV‐related study to incorporate mental health components, ensuring support extends not only to participants but also to their families and partners.

Living with this disease affects more than just your body… it affects your mind, your emotions, your relationships. It's not just the participant who needs mental health support, but their families and partners, too.—Male, White

Suggested supports included individual counselling, peer navigation grounded in shared experience and tailored mental health education focused on trauma, stigma and ageing with HIV. Participants underscored that mental healthcare must be an integral part of HIV programmes and policies, implemented through trauma‐informed and person‐centred approaches.

#### 3.2.4 Persistent HIV stigma and discrimination

LTS stressed that HIV stigma remains a pervasive issue, deeply affecting their lives and interactions, particularly in healthcare settings where discrimination still occurs. They called for policies that confront stigma directly through education and awareness to combat the lack of current knowledge about HIV. Some LTS believed a cure might alleviate stigma; however, they also acknowledged that it will not entirely eliminate layers of societal biases about HIV and affected groups, underscoring the need for ongoing efforts to foster HIV understanding and reducing discrimination.

A cure might help with stigma, but it won't erase it all completely. People don't just forget that you had HIV.—Female, White

#### 3.2.5 Medical mistrust and systemic inequalities

Deep‐rooted mistrust in the healthcare system emerged as a pervasive concern among LTS, many of whom described years of being treated as “second‐class citizens” by clinicians uncomfortable with caring for PLWH. This mistrust was compounded for individuals who were also Black, poor or members of the lesbian, gay, bisexual, transgender, queer/questioning, intersex, asexual+ (LGBTQIA+) community, prompting criticisms that the system is designed for those who are “young, healthy, and wealthy” rather than for marginalized populations.

I've been treated like a second‐class citizen in the healthcare system for years. Even now, some doctors are uncomfortable dealing with people who are HIV‐positive. It's hard to trust a system that has failed you so many times.—Male, White

Participants argued that these experiences reflect broader structural inequities that extend far beyond HIV care and erode trust in institutions intended to safeguard public health. Participants highlighted the urgent need for reforms that prioritize transparency, equity and inclusion in healthcare policies and practices, ensuring that all individuals benefit from high‐quality care.

#### 3.2.6 Psychological implications of HIV cure research

LTS expressed deep appreciation for the remarkable progress in ART and a shared hope for an eventual cure. However, many also highlighted the psychological burden of decades of unfulfilled promises related to the HIV cure. They called for heightened sensitivity in how researchers, clinicians and advocates communicate about HIV cure research efforts. A balance between maintaining optimism and acknowledging scientific realities is essential to building community trust. LTS noted that transparent, honest messaging is critical to mitigating the emotional toll of unsuccessful research and ensuring that research‐related information does not promote unreasonable expectations that will give way to repeated disappointment and mistrust.

Cure research is important, but it needs to be communicated in a way that's realistic. Don't promise things you can't deliver. It's not fair to the people who've been waiting for decades.—Male, Black

### 3.3 Social policies and benefits (pre‐cure)

#### 3.3.1 Housing insecurity

Housing insecurity remained a critical and recurring issue for LTS, with many highlighting the compounding effects of ageing, financial instability and medical needs in a landscape of rapidly escalating housing costs. Housing programmes such as Ryan White emergency housing assistance and HOPWA, tailored specifically for PLWH, and the federal Housing Choice Voucher Program (Section 8), designed to assist low‐income families, were considered essential by LTS. However, the potential instability and uncertainty associated with these programmes often led to significant anxiety among LTS.

Housing is going to be a big issue as we get older. Many of us didn't prepare for retirement because we didn't think we'd live this long. The system doesn't account for the fact that HIV is not just a health issue; it's a housing issue, too. Without stable housing, you can't even begin to deal with the other aspects of this disease.—Male, White

Participants emphasized that stable housing is indispensable for addressing the broader health and social consequences of ageing with HIV, urging policymakers to ensure sustained support as LTS confront escalating economic vulnerability.

#### 3.3.2 Economic marginalization

Long‐term financial insecurity was a recurring theme, with LTS describing how decades of living with HIV have left many in precarious economic situations. They called for policies addressing the broader economic challenges faced by LTS.

HIV robbed me of my earning years. I've been on disability for so long that I don't even know how I'd re‐enter the workforce. I feel trapped in poverty, and there's no easy way out. We need programs that help us financially, not just medically.—Transgender Female, Black

Financial insecurity was a pervasive issue for LTS, requiring economic policies that account for the unique challenges of decades‐long survival and limited earning opportunities.

#### 3.3.3 Community building and peer support

LTS underscored the critical role of community‐based programmes in sustaining their wellbeing, describing support groups and peer‐led initiatives as lifelines that provide connection, understanding and safe spaces to share experiences. They called for policies that expand and fund these programmes to combat isolation, address stigma and bolster mental health, particularly for older LTS and those in underserved areas. By ensuring equitable support for these initiatives, policymakers can help reduce isolation, foster resilience and mitigate the enduring psychosocial impacts of HIV, ultimately enhancing the quality of life for LTS as they age.

As we get older, the isolation becomes more real. Many of us don't have families or partners anymore, and society doesn't really care about people like us. We need policies that foster community, not just for young people with HIV but for older survivors, too.—Female, Black

### 3.4 Policy implications in the context of HIV cure (post‐cure)

#### 3.4.1 Safeguarding hard‐won social benefits and programmes

LTS expressed apprehension that being declared “cured” could jeopardize essential benefits like housing, healthcare and disability services. Their concerns were underscored by Timothy Ray Brown's case, where he reportedly lost HIV‐specific housing assistance post‐cure. Participants stressed that HIV fundamentally alters life trajectories, and removing the virus does not restore full health or workforce readiness after decades of ART‐related impacts.

If I'm cured but still dealing with all the damage HIV has done to my body, will I lose my benefits? I can't work like I used to, and I depend on those programs to survive. A cure shouldn't erase everything we've been through.—Male, White

They also noted that policymakers often fail to distinguish LTS from newly diagnosed individuals, seeing only HIV status. LTS called for structural protections and transitional policies to prevent abrupt loss of support and ensure they are not forgotten once cured.

#### 3.4.2 Equity in access to cure

LTS emphasized that equity must be central to all HIV cure‐related policies. They stressed that cure interventions should be accessible and affordable for everyone, including people from racially and ethnically diverse groups, with low income, and LTS. Without equity, they argued, cure strategies or interventions may become yet another source of exclusion rather than a reason for hope.

If the cure is only for people who can afford it, it's not really a cure. It needs to be available to everyone, especially those who've been most affected by this disease, people of color, low‐income folks, and long‐term survivors.—Male, White

### 3.5 Broader structural reforms and future policy directions

#### 3.5.1 Workforce re‐entry challenges

LTS faced substantial barriers to re‐entering the workforce after decades of illness‐related unemployment. Outdated disability policies, the lack of late career job opportunities and ageism in hiring practices contributed to economic insecurity. Many LTS expressed concern about losing critical benefits such as disability income, housing assistance and healthcare coverage if they attempt to work, creating a systemic “benefits cliff” that discourages employment.

Loss of benefits is always… like right now. People are feeling better, like I said, and they're going back to work. But their concern is: ‘Am I going to earn too much money where it's going to cause me to lose my benefits?—Male, White

LTS urged that policies must address these structural barriers through tailored job training, flexible work arrangements and protections against the loss of essential benefits. Without such reforms, LTS remained trapped in a cycle of economic insecurity, perpetuated by systems that were initially designed to support short‐term disability.

#### 3.5.2 HIV cure research funding and ethical oversight

Participants voiced concerns about the lack of urgency in HIV cure research funding compared to other diseases, such as COVID‐19.

It feels like HIV isn't a priority anymore. We saw how quickly they developed a vaccine for COVID. Why hasn't that same urgency been applied to finding a cure for HIV? It's frustrating because we've been waiting for decades, and it feels like we've been left behind.—Male, Black

They also highlighted the need for ethical oversight to protect potentially vulnerable populations and ensure transparency. LTS emphasized that greater investments in HIV cure research are essential, accompanied by rigorous ethical standards to ensure transparency and protect the rights of participants.

#### 3.5.3 Inclusion in policy and research decisions

LTS expressed frustration about being excluded from policymaking and research decisions, emphasizing the need for their voices to be heard in shaping HIV‐related policies.

Representation matters… If long‐term survivors aren't part of the conversation, the policies won't reflect our reality.—Male, White

Inclusion of LTS in policymaking and research designs was seen as essential to ensuring that their lived experiences inform decisions and that policies address their actual needs.

## 4 DISCUSSION

This study provides important insights into the policy priorities of LTS of HIV in the United States, highlighting both current challenges and potential future concerns should a cure become available. We identified four urgent policy domains. First, fragmented Medicare and Medicaid coverage impedes access to essential services, particularly mental health, dental and treatment for comorbidities, resulting in disproportionate financial burdens on LTS. Second, pervasive stigma and discrimination in healthcare persist, eroding trust in clinical services and compounding the marginalization of individuals who also face biases related to race, socio‐economic status and/or sexual identity. Third, stable housing and economic support programmes remain critical for healthy ageing. Finally, fears of losing vital support services upon being deemed “cured” dampen enthusiasm to participate in cure research, underscoring the need for transitional policies that ensure continuity of care. These findings reinforce calls from initiatives like the San Francisco Principles [25] for equitable healthcare, inclusion and mental health support for LTS.

LTS have effectively outlived the policy frameworks established during earlier phases of the epidemic. Although ART has significantly extended survival, many LTS suffer accelerated/accentuated ageing plus multiple HIV‐associated comorbidities, leaving them to face high copays, limited coverage for mental health and dental care, and bureaucratic barriers [26, 27, 28, 29]. While some challenges are shared with the broader HIV population, LTS face them with unique intensity due to decades of trauma, early toxic treatments and ageing‐related burdens [2, 30, 31]. These long‐term exposures position LTS as a unique sub‐population whose lived experiences must inform responsive and forward‐looking policy agendas.

Equally central to LTS’ wellbeing is addressing social determinants of health. Although programmes such as HOPWA and Section 8 or the Housing Choice Voucher Program (which provides rental subsidies to low‐income individuals and families) help reduce housing insecurity, ongoing concern around funding and eligibility often compounds anxiety [9, 10]. The Ryan White HIV/AIDS Program, a critical funding source for medical and support services, faces persistent budget challenges, with recent shortfalls jeopardizing core services [32]. Recent shifts in federal policy and fluctuations in public health funding have further strained critical support systems for PLWH, including LTS, exacerbating challenges related to healthcare access, housing stability and economic security. Moreover, decades of interrupted employment and outdated disability policies have left many LTS financially vulnerable [1, 33]. Within this uncertain landscape, peer‐led and community‐based initiatives can serve as powerful protective factors, helping to alleviate social isolation and sustain resilience [34]. If these overlapping structural needs remain unmet, the public health implications are substantial. Unaddressed mental health challenges, housing instability and chronic medical conditions may contribute to increased healthcare utilization, premature hospitalization and worsening comorbidities, placing greater financial strain on healthcare systems and limiting the capacity to engage older adults in future HIV prevention and cure initiatives [35, 36].

The prospect of effective curative interventions for HIV introduces an additional layer of complexity with the potential loss of essential support services [2, 14, 19]. These concerns underscore the need for updated community education programmes to address such concerns and the necessity of transitional policies that preserve essential safety nets while acknowledging the cumulative physical and psychological burdens LTS continue to bear. In parallel, equitable access to future cure interventions is paramount to avoid deepening disparities [34, 37]. LTS also emphasized that a cure alone would not erase the profound trauma and stigma endured over decades, calling for transparent communication and psychosocial support to manage expectations [1, 19, 38, 39].

Collectively, these findings highlight the urgent need for integrated policy reforms that address both the clinical and structural realities of ageing with HIV [40]. Possible strategies include systematically embedding mental healthcare into HIV services; strengthening housing, economic protections and support services; and establishing ethical guidelines that safeguard LTS’ rights and wellbeing in the event of an HIV cure. Crucially, LTS themselves must be central collaborators in shaping these measures, to shape stigma reduction, peer support and research design. Although this study focused on the United States, many of these challenges are echoed globally, underscoring the need to include LTS voices in international HIV cure research and policy development. By addressing LTS‐specific needs and shared challenges with the broader HIV community, public health systems can reduce long‐term costs, build resilience and ensure that no segment of the HIV community is left behind. In addition to identifying these intersecting priorities, participants articulated a range of concrete policy recommendations aimed at improving the long‐term wellbeing of LTS. These include protections for benefits, and investment in trauma‐informed care, housing and mental health. Table S3 provides a detailed summary of these participant‐informed policy considerations, which can serve as a roadmap for researchers, policymakers and programme developers working towards equitable HIV cure readiness.

This study has notable limitations. Recruitment through CBOs may have introduced sampling bias, and we were unable to ascertain the total number of potential candidates reached. Despite racial and gender diversity, Hispanic individuals were not represented, reflecting the ongoing underrepresentation of Spanish‐speaking PLWH in U.S. research. Additionally, as all participants were based in the United States, the generalizability of our findings to other contexts remains uncertain. Because experience in HIV advocacy or peer engagement was part of the inclusion criteria, the sample likely reflects a more informed and civically engaged segment of the LTS population, whose views may differ from those of less‐engaged individuals. While our focus was on older LTS, the unique experiences of lifetime survivors living with HIV from birth or early childhood represent a critical area for future exploration.

## 5 CONCLUSIONS

LTS have faced unique, evolving challenges that demand immediate policy action. This study emphasized the urgent need for government, academia and industry to address ageing‐related comorbidities, integrate mental healthcare and mitigate HIV stigma. Equitable access to housing, financial stability and community support is essential to enhancing LTS’ quality of life pre‐ and post‐cure. As HIV cure research efforts advance, policies must proactively safeguard the continuity of essential services and protect the health and social benefits of all PLWH, including ageing populations that comprise over 50% of PLWH in the United States. Failure to do so risks exacerbating disparities and undermining the progress of HIV cure research itself. Incorporating LTS in policymaking and research is crucial to addressing systemic inequities, ensuring a more resilient and equitable public health framework that benefits LTS and all PLWH.

## COMPETING INTERESTS

KD provides advisory services to Gilead Sciences, Inc. AbbVie, Inc. and Viiv Healthcare, Inc. MK reports historical funding to the institution from Gilead Sciences and ViiV Healthcare. LD reports historical AIDS Action Baltimore funding from ViiV Healthcare. JB provides advisory services to AbbVie, Gilead Sciences, Merck and Co., and ViiV Healthcare, and reports organizational funding from Gilead Sciences and ViiV Healthcare. JT provides advisory services to Abbvie and ViiV Healthcare, and receives organizational funding from Gilead Sciences & ViiV Healthcare. DA provides advisory services to Merck and Co, Gilead Sciences and ViiV Healthcare, and The Well Project receives organizational funding from Gilead Sciences, Merck and Co, and ViiV Healthcare.

## AUTHORS’ CONTRIBUTIONS

KD, JT and LD were involved in conception and design; KD and JT were involved in the collection of data; JC‐JL, RL and SDD reviewed each transcript for accuracy and completeness and compared them with the corresponding audio files. AA and KD were involved in the analysis and interpretation of the data, and AA drafted the paper. All co‐authors reviewed it for intellectual content, approved the final version of the manuscript and agreed to be accountable for all aspects of the work.

## FUNDING

This study was funded by a community‐based grant shared between RID‐HIV v1.0 (UM1 AI164561), DARE v3.0 (UM1 AI164560) and CRISPR for Cure v1.0 (UM1 AI164568), administered through AIDS Action Baltimore. KD contributed separate support from the BEAT‐HIV (UM1AI164570) and R01MH126768 to facilitate data analysis and manuscript development for this project. The RID‐HIV, DARE, CRISPR for Cure and BEAT‐HIV Martin Delaney Collaboratories for HIV Cure Research are supported by the following U.S. NIH Institutes and Centres: NIAID, NHLBI, NIDDK, NIDA, NIMH and NINDS. We also acknowledge support received from P01 AI169609 (Smith—Leaving, Coming and Staying HIV Obligate Microenvironments (HOME)). ML is supported by the University of Washington/Fred Hutch Center for AIDS Research (CFAR), an NIH‐funded programme under award number AI027757, which is supported by the following NIH Institutes and Centres: NIAID, NCI, NIMH, NIDA, NICHD, NHLBI, NIA, NIGMS and NIDDK. MK is supported by R01 MH134261. This research was also supported by the San Diego Center for AIDS Research (SD CFAR), an NIH‐funded programme (P30 AI036214), which is supported by the following NIH Institutes and Centres: NIAID, NCI, NHLBI, NIA, NICHD, NIDA, NIDCR, NIDDK, NIMH, NIMHD, NINR, FIC and OAR.

## REPRINT REQUESTS

Please direct to: kdube@health.ucsd.edu

## Supporting information




**Table S1**: Institutional Review Board‐Approved Interview Guide Exploring Policy Priorities Among Long‐Term Survivors of HIV in the United States, 2023–2024
**Table S2**: Additional Quotes – Policy Priorities Suggested by LTS in the Context of Research
**Table S3**: Policy Considerations Suggested by LTS in the Context of Research Towards an HIV Cure

## Data Availability

The original contributions presented in this study are included in the main body of manuscript and supplementary materials. Further inquiries can be directed to the corresponding author.
