# Supplementary material for: “A cure might help, but it won't erase it all”: a qualitative study of policy challenges and priorities for long‐term survivors of HIV in the United States
Source: J Int AIDS Soc. 2025 Jul 3;28(7):e70006. doi: 10.1002/jia2.70006 (PMC12231189; doi:10.1002/jia2.70006)
Supplement: Supplementary file 1 — Table S1: Institutional Review Board‐Approved Interview Guide Exploring Policy Priorities Among Long‐Term Survivors of HIV in the United States, 2023–2024 Table S2: Additional Quotes – Policy Priorities Suggested by LTS in the Context of Research Table S3: Policy Considerations Suggested by LTS in the Context of Research Towards an HIV Cure [file JIA2-28-e70006-s001.docx]

**Supplementary Table S1: Institutional Review Board-Approved Interview Guide Exploring Policy Priorities Among Long-Term Survivors of HIV in the United States, 2023–2024**

Thank you for participating in this interview. This study will help us understand how we can better engage long-term survivors in HIV cure-related research.

**Introduction**

- - How are you doing today?
  - Can you please tell me/us a little bit about yourself?

**Policy Priorities for long term survivors**

- What should be some of the policy priorities (e.g., current or future issues) for long-term survivors of HIV?
- What should be some of the key issues for the long-term survivor community as it relates to HIV cure research?
  - PROBES: Potential impacts on:
    - Medical benefits (Medicaid/Medicaid)
    - Disability benefits
    - Housing benefits
    - Support groups

**Closing**

- Is there anything else you would like us to know about long-term survivors and HIV cure research related policies that we have not discussed?

Thank you for taking the time to answer these questions. Your participation in this interview significantly contributes to advancing our understanding of how to effectively convey the policy priorities of long-term survivors to policymakers, with the goal of improving their quality of life as they age.

**Supplementary Table S2: Additional Quotes – Policy Priorities Suggested by LTS in the Context of Research**

**Towards HIV Cure**

| **Themes** | **Participant Number** | **Quotations** |
| --- | --- | --- |
| **Health Policy Challenges (Pre-Cure)** | | |
| **Healthcare Tailored to LTS** | Male, Black | *I’ve been living with HIV since the 80s, and the medications have kept me alive, but they’ve also taken a toll. My bones are weaker, my heart isn’t as strong, and I have neuropathy in my legs. Doctors treat these as separate issues, but it’s all connected… We need policies that look at the whole person, not just the virus.* |
| **Complex Healthcare Needs and Inadequate Coverage** | Male, White | *We need a much better health care access program or process in this country. There's no reason in this country why everyone doesn't have access to good health care... But for those who are HIV positive, access to quality and competent healthcare is probably the one thing that I would point out.* |
|  | Female, White | *My Medicare Advantage covers most of my costs, but I’ve had to make decisions based on what’s on the list. I can't afford copays. Many people don’t have the knowledge to navigate that.* |
|  | Male, White | *The safety nets are pulled because they’re not prepared for people to get older. Benefits were geared for us to die, not be here 20, 30, 40 years later.* |
| **Mental Health Support** | Male, White | *Trauma-informed care isn’t just a nice idea; it’s essential. Many of us have been through so much, not just the disease but how we’ve been treated by society, by healthcare providers, even by our own families. Policies need to recognize that.* |
| **Persistent Stigma and Discrimination** | Female, White | *A cure might help with stigma, but it won’t erase it all completely. People don’t just forget that you had HIV.* |
|  | Male, White | *I’ve faced discrimination even in healthcare settings. Some doctors don’t want to deal with someone who’s HIV-positive. It’s exhausting to always have to advocate for yourself.* |
| **Medical Mistrust and Systemic Inequalities** | Female, White | *It’s not just about HIV, it’s about being Black, poor, queer, or a combination of those things. The system doesn’t care about marginalized people, and that’s reflected in the way policies are made and implemented.* |
|  | Male, White | *This system isn’t designed for us, its for young, healthy and wealthy.* |
| **Psychological Implications of HIV Cure Research** | Male, White | *I’ve been hearing about the cure being ‘just around the corner’ for 30 years. It’s hard to stay hopeful when you’ve been disappointed so many times. We need honesty, not hype.* |
|  | Female, White | *Hope is a double-edged sword. I want to believe a cure is coming, but I’ve been burned before. It’s emotionally draining to keep getting your hopes up only to be let down.* |
| **Social Policies and Benefits (Pre-Cure)** | | |
| **Housing Insecurity** | Male, White | *Housing is going to be a big issue as we get older. Many of us didn’t prepare for retirement because we didn’t think we’d live this long. The system doesn’t account for the fact that HIV is not just a health issue; it’s a housing issue, too. Without stable housing, you can’t even begin to deal with the other aspects of this disease.* |
| **Economic Marginalization** | Male, White | *Many of us didn’t plan for the future because we didn’t think we’d live this long. Now we’re here, but we’re broke and can’t get the help we need. The system doesn’t account for what it means to survive.* |
| **Community Building and Peer Support** | Male, White | *Support groups can be really important for getting out and mingling with other people, especially when you’re 65 years old. And if you don’t have a lot of friends, it gives you things to do outside of just being home all the time.* |
|  | Male, Black | *At this time, a support group would be a wonderful experience to have, especially when you’re living with the day-to-day issues of HIV and aging.* |
|  | Female, Mixed race | *I’ve outlived so many friends. Sometimes I feel like I’m the last one left. We need to create spaces where survivors can connect, share, and feel less alone.* |
|  | Male, White | *For me, my support group has been a lifeline. It’s not just about talking about HIV—it’s about finding people who understand what it’s like to live this life. Without that community, I think I would’ve given up years ago.* |
|  | Male, White | *We need more of this. Support groups are huge. We need better access to them. I mean, I don’t live in the middle of the United States, but I would be surprised if they have the support groups that places like Philadelphia offer.* |
| **Policy Implications in the Context of HIV Cure (Post-Cure)** | | |
| **Safeguarding Hard-Won Social Benefits and Programs** | Female, Black | *If they [researchers] decide I’m ‘cured’ one day and say I don’t need HOPWA anymore, what happens to me? Housing is not just about HIV. It’s about being older, being disabled, and being vulnerable in a system that doesn’t care about people like me.* |
|  | Female, White | *Programs like HOPWA and Ryan White have kept me alive. If those go away after a cure, where does that leave us? Policies need to guarantee that we won’t lose what we have.* |
|  | Transgender Female, Black | *Being cured doesn’t mean I’m suddenly healthy or able to work. We need transitional policies that support people through the process, not just cut them off.* |
|  | Male, White | *If you’re HIV positive... and you become cured, and you haven't worked in 15 years because you haven't had the health to work... You could end up homeless and, on the street, very quickly.* |
|  | Female, White | *Timothy Ray Brown lost his housing benefit when he became cured in Palm Springs. They kicked him out because he didn’t qualify anymore without HIV.* |
| **Equity in Access to Cure** | Female, White | *Equity has to be at the center of any policy about the cure. If it’s not affordable or accessible, it’s just another way of leaving people behind.* |
| **Broader Structural Reforms and Future Policy Directions** | | |
| **Workforce Re-Entry Challenges** | Male, White | *Most of us … didn’t prepare for the future. We didn’t think we’d be here. We weren’t planning to work at this age... And now, what job are you going to find when you’re 60 or 70?* |
|  | Male, White | *Even trying to re-enter the workforce, you’re dealing with age discrimination. Employers don’t want to hire people our age, especially if you’ve been out of work for years* |
|  | Male, White | *I had to explain to disability insurance companies why I couldn’t work, and it was so demeaning. ‘Would you rather be a florist or a forest ranger?’ That’s the kind of nonsense we had to go through.* |
|  | Female, White | *I live on such a tight budget that any unexpected expense feels like a crisis. A cure won’t fix my financial situation. We need policies that address the economic realities of being a long-term survivor.* |
| **HIV Cure Research Funding and Ethical Oversight** | Female, White | *Funding for HIV cure research is critical, but it needs to be done ethically. Vulnerable people, like long-term survivors, shouldn’t be exploited or used as guinea pigs.* |
|  | Female, Mixed race | *The research is important, but we’ve been burned before. People sign up for trials out of desperation, hoping for a cure, but they don’t always know what they’re getting into. Policies need to make sure people are fully informed and protected.* |
|  | Female, White | *Researchers need to be honest about the risks and not overpromise.* |
| **Inclusion in Policy and Research Decisions** | Male, White | *Researchers and policymakers talk about us but rarely talk to us. We need to be at the table when these decisions are made because no one knows our needs better than we do.* |
|  | Female, Mixed race | *We’ve been living with this disease for decades. No one understands it better than we do. But when it comes to making decisions, our voices are often ignored. Policies need to include us, not just as participants but as decision-makers.* |

LTS: Long term Survivors

**Supplementary Table S3: Policy Considerations Suggested by LTS in the Context of Research Towards an HIV Cure**

| **Policy Domains** | **Policy Considerations** |
| --- | --- |
| **Holistic Care Models** | - Develop integrated care models combining HIV management with geriatric, mental health, and social care services. - Introduce patient-centered approaches that empower LTS to participate in care planning. - Expand telehealth options to increase access to specialized care for LTS in rural or underserved areas. - Ensure continuity of care for LTS transitioning between HIV and post-cure healthcare systems. |
| **Healthcare Access** | - Reform Medicare and Medicaid to address gaps in coverage for aging-related comorbidities, including cardiovascular disease, osteoporosis, central nervous system disorders and diabetes. - Ensure coverage for essential services such as dental, vision, and hearing care, which are often excluded but critical for LTS. - Reduce out-of-pocket costs by capping copays and simplifying reimbursement processes. - Establish specialized clinics for integrated HIV and geriatric care to address the unique intersectional needs of LTS. |
| **Mental Health Support** | - Expand access to trauma-informed mental health services tailored to the experiences of LTS, addressing loss, stigma, and long-term stress. - Train healthcare providers to recognize and treat mental health conditions specific to aging individuals living with HIV. - Integrate mental health screenings as a standard part of HIV care protocols. |
| **Stigma Reduction** | - Implement comprehensive anti-stigma campaigns aimed at healthcare providers and the general public. - Develop training modules for medical staff to improve interactions with LTS that reduce implicit biases. - Highlight the contributions of LTS to HIV advocacy through public awareness initiatives. |
| **Housing Stability** | - Ensure sustained funding for programs like Ryan White programs and HOPWA services and expand eligibility to reflect the aging HIV population. - Develop long-term housing solutions that include supportive services for older adults with HIV, such as assisted living and transitional housing. - Address systemic barriers to housing access practices. - Expand Ryan white housing stability grants for LTS facing financial emergencies. |
| **Economic Stability** | - Revise disability and retirement policies to account for the interrupted work histories of LTS, enabling financial stability as they age. - Provide financial planning and counseling programs to help LTS manage long-term financial challenges. |
| **Community Building and Support** | - Expand Ryan White funding for peer-led support groups and community centers specifically designed for aging LTS. - Foster social connection programs that include regular events, skill-sharing initiatives, and mentorship opportunities. - Increase investment in virtual community-building platforms to support geographically isolated LTS. - Partner with local organizations to create inclusive spaces that combat stigma and loneliness. |
| **Workforce**  **Re-entry** | - Develop age-inclusive job training programs for LTS to build skills and confidence for re-entering the workforce. - Protect and expand essential benefits, such as Ryan White support services and Medicaid benefits, to prevent a "benefits cliff" for those returning to work. - Create local employment incentives for businesses to hire older adults living with HIV. - Support alternative work arrangements, such as part-time roles or remote jobs, to accommodate the health needs of aging LTS. |
| **HIV Cure Policy** | - Design transitional policies to maintain benefits for LTS during and after being declared “cured.” - Ensure equitable access to an HIV cure by prioritizing low-income individuals, racially and ethnically diverse communities, and marginalized groups disproportionately affected by HIV. - Protect LTS from losing housing, medical, and Ryan White social services upon reclassification of HIV status post-cure. - Fund educational campaigns to inform LTS about potential benefits and risks of cure-related interventions. |
| **Ethical Research Practices** | - Ensure the inclusion of LTS in the design and oversight of HIV cure research. - Establish ethical guidelines to prevent exploitation of populations during research trials. - Provide comprehensive informed consent processes that explain risks, benefits, and long-term implications of participation in HIV cure research. - Include monitoring provisions for participants in cure trials to identify long-term adverse events and any emerging health issues. - Prioritize funding for studies that identify short and long-term adverse events related to cure interventions and clinical trial design strategies. |
| **Funding and Investment** | - Advocate for increased federal and state investment in HIV aging programs, including mental health, housing, and workforce initiatives. - Create grant programs to support grassroots organizations serving LTS. - Prioritize funding for long-term studies examining the aging process and comorbidities among LTS. - Ensure sustainability of critical HIV programs through multi-year funding commitments to prevent gaps in services. |

LTS: Long term Survivors
